# The Impact of Light Spectrum and Intensity on the Growth, Physiology, and Antioxidant Activity of Lettuce (*Lactuca sativa* L.)

**DOI:** 10.3390/plants10102162

**Published:** 2021-10-12

**Authors:** Shiren J. Mohamed, Hail Z. Rihan, Naofel Aljafer, Michael P. Fuller

**Affiliations:** 1 School of Biological and Marine Sciences, University of Plymouth, Plymouth PL4 8AA, UK; hail.rihan@plymouth.ac.uk (H.Z.R.); naofel.aljafer@plymouth.ac.uk (N.A.); mfuller@plymouth.ac.uk (M.P.F.); 2 Biotechnology and Crop Sciences Department, College of Agricultural Engineering Sciences, University of Sulaimani, Sulaimani 46001, Iraq; 3 Phytome Life Sciences, Launceston PL15 7AB, UK

**Keywords:** LEDs, lettuce, physiology, fresh weight, antioxidant activity

## Abstract

This study focused on the physiology, growth and antioxidant activity response of hydroponically grown lettuce (*Lactuca sativa* L.) under sole-source LED lighting of differing spectra. Lighting spectra were provided by differing combinations of LEDs of three different peak wavelengths, (Blue 435, Blue 450, and Red 663 nm) with ratios of B450/R663: 1.25 ± 0.1, B450/R663: 1.25 ± 0.1, and B450/R663 1:1 at two light intensities of photosynthetically active radiation (PAR) (270 μmol m^−2^ s^−1^ and 60 μmol m^−2^ s^−1^). A further experiment was conducted, in which Blue and Red LEDs were supplemented with Green (Blue 450, Red 663, and Green 520 nm) with ratios of B435/R663: 1.25 ± 0.1, B450/R663/G520: 1/0.73/0.26, and B450/R663: 1.25 ± 0.1. LED light intensities under the different spectra were adjusted to deliver the same level of PAR (270 ± 20 μmol m^−2^ s^−1^). Results from the first experiment showed that increased fraction of blue 435 nm in combination with red light at 663 nm at high irradiance enhanced the physiology of lettuce (i.e., significantly increased assimilation rate, stomatal conductance and transpiration rate) and increased the yield while having no significant effect on antioxidant activity. At the lower irradiance, the B435/R663 significantly increased antioxidant activity compared to other spectra. Results from the second experiment showed no significant effect of the spectra of LEDs on the physiology and yield of lettuce, but antioxidant activity was very significantly induced by B450/R663 at the ratio of 1.25 ± 0.1. However, the amount was still less than that obtained by B435/R663 1.25 ± 0.1 from the first experiment. This study indicates that LED light with a spectrum of B435/R663 at a ratio of 1.25 ± 0.1 significantly improves lettuce yield and antioxidant activity.

## 1. Introduction

Lettuce (*Lactuca sativa* L.) belongs to the family Composite, and is an important dietary leafy salad vegetable that is primarily consumed fresh or in salad mixes [1]. It is a major source of bioactive compounds with diverse biological activities: it has antioxidant, anti-inflammatory, anticancer, antimicrobial, cholesterol lowering, and antidiabetic effects, and it is a good source of fibre, iron, folate, and vitamin C [2,3,4,5]. Lettuce is widely grown in semi-controlled environments in glasshouses and plastic tunnels, often using hydroponic culture [6,7]. Some lettuce crops are now grown under controlled conditions using artificial light in plant factories. Lettuce is frequently used as a test species when investigating the optimisation of plant factory conditions.

Light is one of the fundamental environmental factors for plant growth and development. Light quality, in comparison with light intensity and photoperiod, has been shown to have a much more complex impact on plant physiology and morphology in terms of spectral distribution, since specific wavelengths stimulate different physiological and morphological responses [8] This indicates the importance of chlorophyll A&B. Chlorophyll A is the primary pigment of photosynthesis and absorbs light from 430 nm to 662 nm. Chlorophyll A has a central role in the transference of energy to the reaction centre and contributes very significantly to the electron transport chain, since it donates two excited electrons. Chlorophyll B absorbs a blue light range between 453 nm to 642 nm, chlorophyll B helps organisms to convert the energy from light spectra to chemical energy. Furthermore, chlorophyll B can absorb a wider range of wavelengths of light, which enables more energy to be transferred to chlorophyll A [9].

Light-emitting diodes (LEDs) have higher luminous efficiency, long life, and higher efficacy, leading to reduced associated heating [10] compared to other artificial lighting sources, such as fluorescent bulbs or sodium vapour lamps. Furthermore, in indoor plant factory farming systems, LEDs allow for the modification of the spectrum to fit the plant species requirements. Lettuce is widely cultivated in plant factories under LEDs [11,12], because of its adaptability to controlled environments, its short growth cycle, and defined rosette shoot shape [13]. Plant Factories (controlled environment agriculture) are new forms of agriculture that are not dependent on arable land and that can be developed in the urban environment are gaining increasing popularity. Plant Factories with Artificial Lighting (PFALs) or Vertical Farms with Artificial Lighting (VFALs), are closed plant production systems where environmental factors (e.g., temperature, humidity, light, and CO_2_ concentration) are controlled, minimizing the interactions with the external climate. There is a significant growing interest in this form of farming because it can be built anywhere, high resource use efficiency (water, CO_2_, fertilizer, etc.) can be achieved with minimum emission of pollutants to the outside environment, the growing environment is not affected by the outside climate and soil fertility, production can be year-round and productivity is over 100 times that of field production, produce quality such as concentrations of phytonutrients can be enhanced through manipulation of the growing environment, especially light quality; produce is pesticide-free and need not be washed before eating; produce has a longer shelf life because the bacterial load is generally less than 300 CFU g^−1^, which is 1/100 to 1/1000 that of field-grown produce and energy for transportation can be reduced by building PF near urban areas. Light is a key factor and a very important element for the Plant Factories since it has direct impact of growth, yield, and quality of plants.

It has been reported that lettuce grown under combined Red and Blue LEDs exhibit the highest chlorophyll content, photosynthesis rate [14], pigment content, leaf numbers, leaf area index and shoot dry weight, and also increased antioxidant activity [15]. However, plants under monochromatic Blue or Red LEDs have displayed growth abnormality and reduced photosynthetic rate [14,16]. Recently, Naznin et al. [15] reported that lettuce grown solely under Red LEDs had significantly reduced biomass, chlorophyll content, carotenoid content, and antioxidant levels. Moreover, it has been observed that the lettuce plants could not perform normally in Red light only, and the combination of 90% Red and 10% Blue was considered more effective [17]. Photosynthesis rate, stomatal density, growth, and mineral element content under a combination of Red and Blue appears to be dependent on the Red light/Blue light ratio (R/B ratio and all these parameters increased with a decrease in R/B ratio) [14]. Pennisi et al. [10] reported that, when the R/B ratio increased from 0.5 to 3, the chlorophyll and flavonoid content, nutrient uptake and water use efficiency of the lettuce leaves improved, with a resultant yield increase of 1.6-fold, although no further increase was reported when the R/B exceeded a ratio of 3. It has been reported that the optimal ratio of R/B for lettuce is at an intensity of 200 µmol m^−2^ s^−1^ irradiance for 16 h for highest photosynthesis rate and stomatal conductance is R/B = 1 compared to ratios of R/B of 4, 8, 12 with a significant decrease when the ratio of R to B increased from 1 to 12 [14].

The synergistic effectiveness of the combined Red to Blue ratio can be more clear on lettuce growth in term of leaf area and dry weight when a small quantity of green G light (24%) is added, since green light is better able to penetrate the plant canopy than Red or Blue light [18]. This may be because the plants have sensitive green light sensors (phytochromes and cryptochrome), although their efficiency in processing green is less than that shown in response to blue and red wavelengths [19]. In contrast, Saito et al. [20] reported that lettuce plants under monochromic Red light had a higher photosynthetic rate, greater leaf number and greater fresh weight compared to either blue light or a mixture of RB light. These findings are supported by Wang et al. [14], who concluded that Red light might be the most effective wavelength for photosynthesis and growth in lettuce (*Lactuca sativa* L.). Lee and Kim [21] also concluded that Red light LEDs with a peak of 634 nm and 659 nm and Blue light LEDs with a peak of 450 nm are the potential spectral wavelengths that boost the photosynthetic rate most effectively, leading to increased leaf area, shoot fresh weight, leaf chlorophyll, and anthocyanin content.

However, there is less agreement regarding optimal fraction of either R or B combination effect on lettuce. The current study therefore aimed to investigate the different fraction of R and B LEDs and different RB ratios on the physiology, growth and antioxidant activities in lettuce (*Lactuca sativa* L.).

## 2. Materials and Methods

### 2.1. Plant Material and Growth Condition

Lettuce seeds were obtained from CN seeds (CN Seeds Ltd., Pymoor, UK), then sown and germinated in the greenhouse at Skardon Gardens. When seedlings had their first pair of true leaves, they were transferred to the plant factory facility at the University of Plymouth. The university’s plant factory facility is a converted insulated greenhouse where external light has been excluded. The multi-tier hydroponic growing system consists of gulleys for NFT (nutrient film technique) and is installed with interchangeable LED light units. The plant factory is divided into several multi-shelf hydroponic units, each consisting of three tiers. The distance between tiers is 50 cm, and 16 plants were planted in each tier at a spacing of 20 cm within a gully and 20 cm between gullies. The temperature and humidity were monitored, using Gemini data loggers (Tinytag Plus (part No GP-1590)) and an instantaneous thermometer (Fisher Scientific, Hampton, NH, USA) at 23 ± 2 °C. The light/dark period was set to 16/8 h.

Two experiments were established:

#### 2.1.1. First Experiment

Three lighting treatments were designed and applied at two intensities (high: 270 and low 60 μmol m^−2^ s^−1^) measured using a UPRtek MK350N premium Standalone handheld spectral light meter, Taiwan. Light treatments were as follows:

Blue 435 nm rich treatment: Blue/Red (B435/R663): Blue rich spectrum with 435 nm wavelength used as a source of blue (B/R: 1.25 ± 0.1) (Blue 435 nm to Red 663 nm spectrum peak ratio, 1.6:1).

Blue 450 nm rich treatment: Blue/Red B450/R663: Blue rich spectrum with 450 nm wavelength used as a source of blue (B/R: 1.25 ± 0.1) (Blue 450 nm to Red 663 peak ratio, 1.6:1).

**Figure 1:**
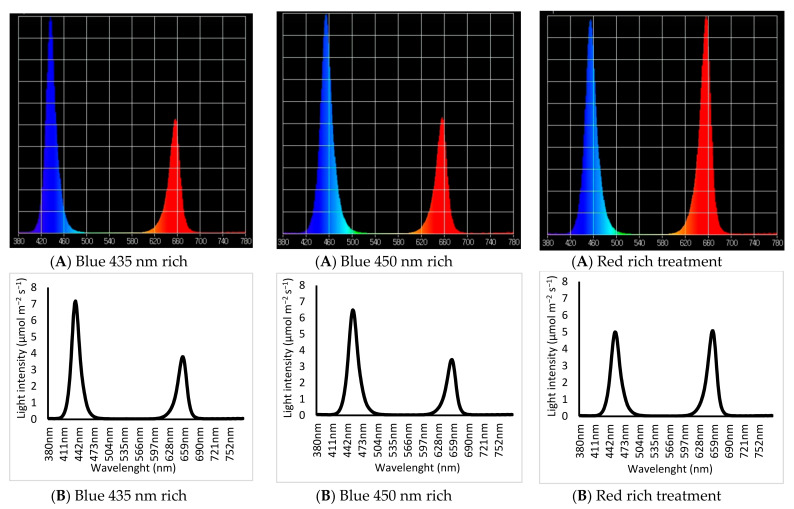
Spectra of the LED treatments (Blue 435 nm rich treatment, Blue 450 nm rich treatment and Red rich treatment), as measured by an UPRtek spectrophotometer: (**A**) the relative light intensity. (**B**) The radiant density of the light spectrum intensity.

**Figure 2:**
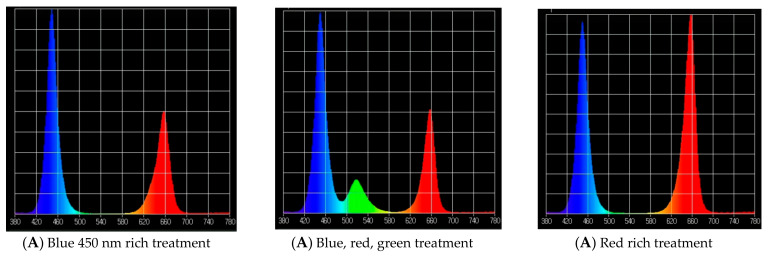

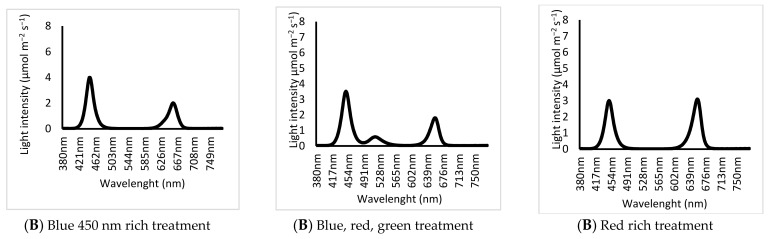
Spectra of the LED treatments (Blue/Red treatment (B-rich/R), blue, red, green treatment and red rich treatment) as measured by an UPRtek spectrophotometer: (**A**) the relative light intensity. (**B**) The radiant density of the light spectrum intensity.

Red rich treatment: Blue/Red treatment (B/R-rich): Red 663 nm rich light spectrum with 450 nm wavelength used as a source of blue (B/R: 0.72) Blue to Red ratio, (1:1). (Figure 1 and Figure 2).

#### 2.1.2. Second Experiment

Three lighting treatments at 170 ± 10 μmol m^−2^ s^−1^ were designed as follows:

(1) Blue 450 nm rich treatment: Blue/Red treatment (B-rich/R). Blue rich spectrum with 450 nm wavelength used as a source of blue (B/R: 1.25 ± 0.1) (Blue (450 nm) to Red (663) peak ratio, 1.6:1).
(2) Blue, red, green treatment: Blue/Red/Green treatment (B/R/G). Blue rich spectrum with 450 nm wavelength used as a source of blue with (B/R/G: 1.25/1/0.35) (Blue (435 nm) to Red (663) peak ratio, 1.6:1).
(3) Red rich treatment: Red (663 nm) rich light spectrum with 450 nm wavelength used as a source of blue (B/R: 0.72) (Blue Red peak ratio 1:1.2).

### 2.2. Physiological Parameters Measurements

Physiological response (assimilation rate μmol m^−2^ s^−1^, stomatal conductance mmol m^−2^ s^−1^, and transpiration rate mmol m^−2^ s^−1^) of planted lettuce to the lighting treatments was measured at two stages of development: at the initial vegetative, stage 4 weeks from the transplanting of plantlets; and the second (final) harvest stage, conducted 7 weeks after transplanting plantlets to the plant factory setting. The three unfolded top leaves were chosen from five plants from each treatments. Physiological measurements included light-saturated instantaneous maximum photosynthetic rate Amax (μg cm^−2^ s^−1^) was measured using an LCi-SD Highly Portable Ambient Photosynthesis System (ADC BioScientific, Herts, UK).

### 2.3. Determination of Plant Morphology

Morphological response of planted lettuce to the lighting treatments were measured at two stages of development: at the initial vegetative, stage 4 weeks from the transplanting of plantlets; and the second (final) harvest stage, conducted 7 weeks after transplanting plantlets to the plant factory setting. Morphological measurements of five randomly chosen plants from each treatment were taken. These included leaf number (cmshoot fresh weight (FW); and root fresh weight (RFW) (g), using a sensitive Fisher Scientific SG-402 laboratory balance.

### 2.4. Antioxidant Activity Analysis

The plants (all plants) from the second cut were stored in a deep freezer (at −20 °C) and freeze-dried for antioxidant analysis. The total antioxidant activity was analysed using the Ferric Reducing Ability of Plasma (FRAP) assay [22]. The method is based on the reduction of Fe^3+^ TPTZ complex (colourless complex) to Fe^2+^-tripyridyltriazine (blue coloured complex) formed by the action of electrons donating antioxidants at low pH. This reaction was monitored by measuring the change in absorbance at 595 nm. The Ferric reducing antioxidant power (FRAP) reagent was prepared by mixing 10 part of 300 mM acetate buffer, 1 part of 10 mL TPTZ in 40 mM HCl and 1 part 20 mM FeCl_3_·6H_2_O. For the extraction, 0.1 g of freeze dried leaves were weighed and ground using a mortar and homogenized with 4 mL of HEPES buffer and sand purified by acid. From this solution, 0.80 mL was placed in an Eppendorf tube and centrifuged at 13,000 rpm in a microfuge (Micro star 12) for 2 min. The extract was then stored on ice prior to use. The calibration curve was prepared by plotting the absorbance at 595 nm versus different concentrations (0, 0.2, 0.4, 0.6, 0.8 and 1 MM) of FeSO_4_. The concentrations of FeSO_4_ were in turn plotted against a concentration of standard antioxidant Trolox. The blank was prepared by mixing the 0.80 FRAP with 0.10 µL HEPES buffer and the spectrophotometer (Bibby Scientific Ltd., Stone, UK) set to zero against the blank. From the each of the stored extract samples, 0.10 µΜ of each stored extract were transferred to the cuvettes and 0.80 mL of FRAP reagent added. The FRAP values were obtained by comparing the absorbance at 595 change in the test mixture with those obtained from increasing concentrations of Fe^3+^ and expressed as mg of Trolox equivalent per gram of sample.

### 2.5. Statistical Analysis

All data were subjected to analysis of variance (ANOVA) using Minitab software (version 17), and comparisons of means were made using the least significant difference (LSD) test at a 5% level of probability.

## 3. Results

### 3.1. Assimilation Rate, Stomatal Conductance and Transpiration at High Lighting Intensity

Assimilation rate in lettuce cultivated under all LED treatments was reduced when the plants reached maturity, (Table 1), which is the assimilation rate at first harvest is significantly (*p* = 0) higher than at second harvest. At both harvest stages, the photosynthesis rate showed a remarkable (*p* = 0.003) difference between LED treatments. At the first harvest, the B435/R significantly increased assimilation rate by 26% compared to the B450/R, while there were no significant differences between B435/R and B/R-rich. At second harvest, there were significant differences between all LED treatments. The B435/R significantly increased the assimilation rate by about 100 and 32% in comparison to B450/R and B/R-rich, respectively. As with the assimilation rate, stomatal conductance in plant leaves grown under all LED treatments significantly (*p* = 0.00) decreased with plants’ maturity (Table 1) and was significantly lower at second harvest compared to first harvest. There was a significant (*p* = 0.00) difference in leaves stomatal conductance between LED treatments at both harvest stages. At the first harvest, the greatest stomatal conductance was under B/R-rich, which increased stomatal conductance by 150 and 400% compared to B435/R and B450/R, respectively. At the second harvest, the greatest stomatal conductance was under B435/R, which was greater by 95 and 290% compared to B450/R and B/R-rich. In contrast to the assimilation rate and stomatal conductance, the transpiration rate increased with plant maturity, (Table 1). Significant (*p* = 0.066) differences between harvest stages were observed, and there was a significant (*p* = 0.004) effect of LED treatments on transpiration rate at both harvest stages. At the first harvest stage, the highest transpiration rate was under B/R-rich, which increased by about 33 and 100% compared to B435/R and B450/R, respectively. At the second harvest stage, the highest value was at B/R-rich, followed by B435/R and then B450/R.

### 3.2. Growth and Morphology at High Lighting Intensity

As shown in Table 1, all LED treatments stimulated lettuce plant growth. Plants produced greater fresh weight (shoot and root) and leaf numbers at second harvest compared to first harvest. At both harvest stages, plants produced different fresh weights (shoot + root) under different LED treatments. Shoot fresh weight (Table 1) was significantly (*p* = 0.003) increased when plants were grown under combination of B435/R, compared to plants grown under B450/R and B/R-rich. At second harvest, the B435/R increased the plants’ fresh weight by 36 and 14% compared to B450/R and B/R-rich, respectively, and the B/R-rich increased plant fresh weight by 13% as compared to B450/R. Furthermore, the highest root fresh weight was produced by plants cultivated under B435/R, compared to other treatments (Table 1). At the second harvest, the B435/R significantly (*p* = 0083) increased the root fresh weight by about 50 and 46% as compared to B450/R and B/R-rich, respectively, and, similarly to shoot fresh weight, the B/R-rich increased the root fresh weight by about 25% compared to B450/R. With regard to the number of leaves, no significant effect between all LED treatments were observed. (Table 1).

### 3.3. Assimilation Rate, Stomatal Conductance and Transpiration at Low Lighting Intensity

All physiological parameters (assimilation rate, stomatal conductance, and transpiration rate) for plants cultivated by all LED treatments with lower light intensity progressively decreased with plant maturity (Table 2), as indicated by a significant reduction of these parameters at second harvest, compared to first harvest. At both harvests, there were significant effects of LED treatment on physiological parameters. Assimilation rate was significantly stimulated (*p* = 0.003) under B 435/R compared to B450/R, with no significant differences between B345/R and B/R-rich, which both increased assimilation rate by 25% compared to B450/R at first harvest. However, at the second harvest, the B435/R increased assimilation rate by 135 and 53% compared to B450/R and B/R-rich, respectively, whereas, at the first harvest, the highest (*p* = 0.072) leaf stomatal conductance was at B450/R, with an increment by 67% compared to other LED treatments. At the second harvest, the highest value of stomatal conductance was obtained by B435/R and B/R-rich, with an increment of 100% compared to B450/R. Although the transpiration rate significantly differed (*p* = 0.003) between LED treatments, the greatest value at the first harvest was under B435/R, with an increment by 60 and 33% as compared to B450/R and B/R-rich, respectively. Furthermore at the second harvest, the greatest value for the transpiration rate was at B345/R, with an increment of about 114% compared with B450/R, and no significant differences between B435/R and B/R–rich were record.

### 3.4. Growth and Morphology at Low Lighting Intensity

In general, plants cultivated by all LED lighting progressively increased growth in term of plant fresh weight (shoot + root), as in Table 2, which shows the shoot weight very significantly greater (*p* ≥ 0.001) and root fresh weight significantly (*p* = 0) greater at second harvest than at first harvest. Plants cultivated at both B435/R and B450/R produced significant (*p* = 0.078) shoot fresh weight (Table 2) which increased shoot fresh weight by 50% compared to B/R-rich at second harvest. Whereas the highest root fresh weight obtained by B 435/R first, then followed by B/R-rich compared to B 450/R (Table 2). Which was at second harvest, the B 350/R increased root fresh weight by about 49 and 22% compared to B450/R and B/R-rich, respectively. There were no significant differences in the number of lettuce leaves grown under different LED treatments (Table 2).

### 3.5. Second Experiment

#### 3.5.1. Assimilation Rate, Stomatal Conductance and Transpiration under B-rich/R, B-rich/R/G and B/R-rich

As in the first experiment, all the physiological parameters; assimilation rate, stomatal conductance, and transpiration rate for plants cultivated by all LEDs progressively decreased when plants reached maturity (Table 3). At both harvest stages, there were no significant (*p* = 0.062) differences between all B-rich/R, B-rich/R/G and B/R-rich lights on the plants’ assimilation rates. Stomatal conductance significantly (*p* = 0.002) has a clear difference between all LEDs treatments, at first harvest B/R-rich increased the stomatal conductance by (20 and 67%) as compared to B-rich/R and B-rich/R/G, respectively. Similarly, the transpiration rate showed significant (*p* = 0.00) differences between LED treatments. The highest values were obtained under B/R-rich, which increased transpiration rate by 94 and 280%, compared to B-rich/R and B-rich/R/G, respectively, at first harvest.

#### 3.5.2. Growth and Morphology under B-rich/R, B-rich/R/G and B/R-rich

Plants grown under all lighting treatment showed an increase in terms of shoots and root fresh weight when plants reached maturity. However, there were no significant (*p* = 0.598) differences between LEDs treatments on plant fresh weight (shoot + root fresh weight) Table 3. Furthermore no significant differences were recorded for plant leaf numbers grown under different LED treatments (Table 3).

### 3.6. Antioxidant Activity

**Figure 3:**
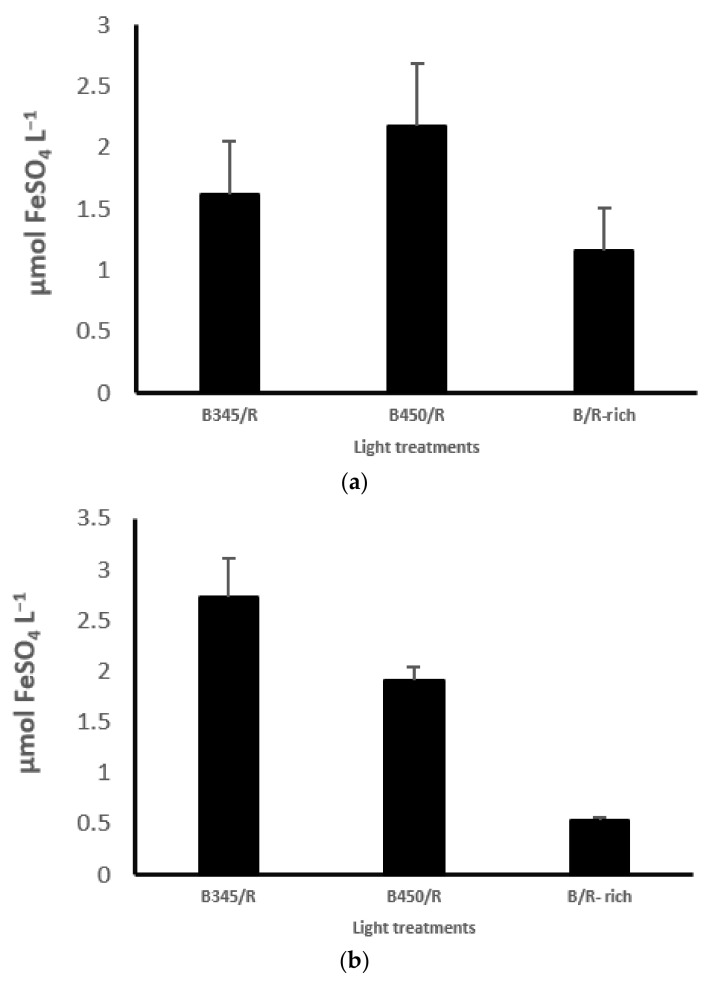

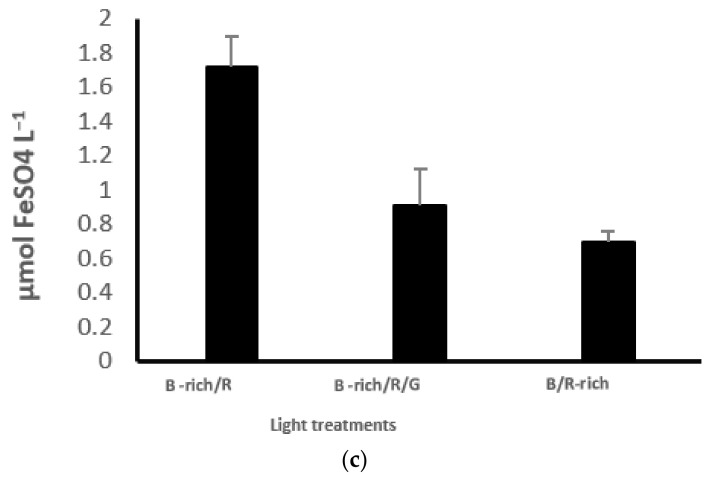
Antioxidant activities in lettuce at second harvest under different LEDs treatments; (**a**) effects of high intensity of B345/R, B450/R and B/R-rich: LSD of treatments = no significance (*p* = 0.296). (**b**) Effects of low intensity of B345/R, B450/Rand B/R-rich: LSD treatments = 0.60 (*p* = 0). (**c**) Effects of B-rich/R, B-rich/R/G and B/R-rich; LSD treatments 0.41 (*p* = 0.004).

At second harvest, there were no significant (*p* = 0.296) differences between all LED treatments with high intensity (270 ± 20 µmol m^−2^ s^−1^). Lettuce shoot antioxidant activity (in µmol FeSO_4_ L^−1^) was recorded, as shown in Figure 3A. The same LED treatments showed a highly significant (*p* = 0) difference in lettuce antioxidant activity (in µmol FeSO_4_ L^−1^) when the intensity of lights became lower 60 µmol m^−2^ s^−1^ (Figure 3B), and the highest level of antioxidant activity (in µmol FeSO_4_ L^−1^) was at B345/R, followed by B450/R, with increasing rate by about 53 and 420%, compared with B450/R and B/R-rich, respectively. In addition, in the second experiment, as shown in Figure 3C, there were significant (*p* = 0.004) differences between LED treatment in lettuce shoot antioxidant activity levels (in µmol FeSO_4_ L^−1^), the highest level was at B-rich/R, with an increment of 125 and 260%, as compared with B-rich/R/G and B/R-rich, respectively.

## 4. Discussion

The results from the first experiment at the higher intensity (270 ± 20 µmol m^−2^ s^−1^) confirmed that, firstly: using blue light with a wavelength peak of 435 nm enhanced assimilation rate by 26 and 100% at first and second harvest, respectively, and this was also observed at the lower intensity (60 µmol m^−2^ s^−1^). The B435/R enhanced assimilation rate by 25 and 135% for the first and second harvest, respectively, more than blue light with a peak of 450 nm. This finding supports our previous study on sweet basil (*Ocimum basilicum*) [23,24]. The second confirmation was that the use of blue light with a wavelength peak of 450 nm could match the absorbance of lettuce pigments when the ratio of B450/R is (1:1) in the balance of LEDs in array increased assimilation rate. In the current study, the stomatal conductance and transpiration rate at the first harvest did not reach the greatest value at B435/R compared to B450/R. At the second harvest, the B435/R gave the highest value of stomatal conductance and transpiration rate with an increment by 95 and 100% of stomatal conductance and by 435 and 114% of transpiration rate, compared to B450/R for first and second intensities, respectively. These findings indicate that the blue light with a wavelength of 435 nm gradually enhanced stomatal conductance, until reaching the highest level at the second harvest.

The third confirmation n was that for lettuce shoot fresh weight, using blue light with wavelength peaks of 435 nm and 450 nm at low light intensity had same effect as that for B435/R and B450/R treatments. The same results for lettuce have been recently reported by [14]: they found that lettuce assimilation rate and fresh weight increased with decreasing R/B ratio from (12 to 1) and this was also associated with an increase in stomatal conductance. This result is in agreement with that of Yan et al. [7], who reported that the assimilation rate in lettuce leaves increased with decrease in R/B ratio. This was due the inhibition of photorespiration and stimulation of stomatal opening to CO_2_ uptake for assimilation [25]. Similarly, increasing in the red light fraction decreased stomatal conductance in lettuce [14] due to the fact that the guard cells of stomata were opened by the blue light phototropin receptors [26]. As a result, plants under blue LEDs maintained photosynthesis more effectively than under red LEDs [27]. Blue (B) and red (R) wavelengths of light are absorbed more by photosynthetic pigments than by other wavelengths. A wide range of plant physiology and growth processes, such as leaf photosynthesis function, photo-morphogenesis, phototropism, stomatal opening [28], hypocotyl elongation, leaf expansion, leaf anatomy, enzyme synthesis, gene expression, and chloroplast movement are driven by blue light [29,30]. On the other hand, red light causes stem elongation and increases chlorophyll content and photosynthesis [31].

Our results are consistent with those of Pennisi et al. [10] who showed that a ratio of R/B = 3 with light intensity of 215 µmol m^−2^ s^−1^ increased chlorophyll content and decreased photosystem II quantum efficiency and transpiration rate, resulting in increased water use efficiency and a maximised lettuce yield. A higher R/B ratio did not result in additional lettuce yield. However, [32] observed that assimilation rate in lettuce decreased as blue light fraction increased from 20% to 30%. The explanation for this is that red light is the most efficient wavelength for photosynthesis. As reported by McCree, (1971), the relative quantum efficiency of red light (600–700 nm) was higher than that of blue light (400–500 nm) because blue fraction was absorbed by flavonoids in vacuoles and/or anthocyanin’s pigments and is less efficient in transferring energy to the reaction centres for photosynthesis [33].

In general, it has been measured at 90% of red and blue light LEDs absorbance by plant [34]. This finding indicates that both red and blue strongly affect plant physiology and development. Plants grown under red LEDs exhibited photosynthesis and growth similar to those grown under blue LEDs [35].

In the current research, the root fresh weight at the low light intensity of 60 µmol m^−2^ s^−1^ under B435/R was higher by 49 and 22% compared to B450/R and B/R-rich, respectively, while both B435/R and B450/R produced the same amount of shoot fresh weight. The reason for this is that increased (“ratio of”?) blue light with wavelength peak of 435 nm to red light with a wavelength peak 663 nm and at a low intensity of LED light can alter the assimilation translocation between lettuce plant organs. A similar tendency was found in lettuce when the ratio of blue to red increased by 20–50% [17].

At both intensities, there was no significant difference in the number of lettuce leaves among plants cultured under all LEDs treatments. In contrast, the combination of R/B LEDs significantly increased leaf numbers in lettuce [14,35,36,37].

In the second experiment, Table 3 shows non-significant differences between all LED treatments B-rich/R (1.6:1), B/R/G (1/0.73/0.26) and B/R-rich (1:1.2) on assimilation rate, while stomatal conductance and transpiration rate significantly increased by B/R-rich, compared to B-rich/R and B/R/G, respectively. This indicated that the supplementation of a small amount of green light to red and blue light could achieve maximum assimilation rate, similar to a combination of red and blue [38]. Moreover, green light can be absorbed by cytochrome (cry), decreasing the activity of chromophores on cry and leading to the induction of stomata opens on leaves by blue light that is absorbed by cry [39]. On the other hand, altering the ratio of blue to red light could be sufficient for lettuce assimilation rate. It is known that the wavelengths of both red and blue lights are necessary in the process of plant photosynthesis [40], indicating that light quantities are more effective than light quality in lettuce production, as recently reported by [7]. As a consequence, the lettuce fresh shoot biomass, root fresh biomass, and leaf numbers did not significantly differ in all LED treatments. These results contradict the findings of Shao et al. [41] who observed that shoot fresh weight in lettuce increased by 20.5% under RBG LEDs with light intensity of 150 µmol m^−1^ s^−1^. This was the result of an increase in the assimilation rate of 24.2% compared to R/B LEDs, because green light is able to penetrate the plant canopy and supply energy, especially in plants with overlapping leaves, such as lettuce. Furthermore, [18] suggested that red and blue LEDs with 24% green treatment gave the highest shoot fresh weight and plants under RBG LEDs and RB had similar assimilation rates. In addition, [42,43] considered the RBG LEDs as an optimal combination wavelength for lettuce growth [44].

In the present study, the levels of antioxidant activity levels in lettuce shoots (in µmol FeSO_4_ L^−1^) under B435/R (1.25 ± 0.1) compared to B450/R (1.25 ± 0.1) B/R-rich (1:1) at the first experiment with low light intensity of 60 µmol m^−2^ s^−1^ but not at the high intensity of (270 ± 20 µmol m^−2^ s^−1^). Moreover, at B/R-rich (1.25 ± 0.1) compared to B/R/G (1/0.73/0.26) and B435/R (1:1.2) at second experiment (Figure 3) were significantly higher than other LED treatments. This was in agreement with Son et al. [45] who reported that increasing the fraction of blue combined with red increased the concentration of phenolic acid and antioxidant activity by 41% at R/B (6:4) and by 24% at R/B (8:2), compared to R/B (9:1) with light intensity of (130 ± 7 µmol m^−2^ s^−1^ 130 ± 7 µmol m^−2^ s^−1^) 12 h photoperiod. The blue light is effective in accumulating secondary metabolism and in promoting the phenolic concentration and antioxidant capacity resulting from an activation of the PAL gateway enzyme in the biosynthesis of phenolic, enhanced by monochromatic blue LEDs [46]. It has frequently been reported that increasing the blue light during light period induced the concentration of many bioactive compounds: this effect has been reported in several cultivars of lettuce [47,48]. More recently, Naznin et al. [15] demonstrated that a higher proportion of blue light to red (83% R + 17% B) compared to (91% R + 9% B and 95% R + 5% B) with intensity of ±200 µmol m^−2^ s^−1^ increased antioxidant activity in lettuce leaves. However, using combined blue and red light led to less change in secondary metabolism and it has been suggested that the metabolic process is more sensitive to change in monochromatic light [45], and the metabolic changes in either monochromatic light or combined light may be induced by differences in the activation of photoreceptors, such as phytochromes and cryptochromes effectively absorbing blue and red light [49].

## 5. Conclusions

The results of this study showed that the growth, photosynthesis, and antioxidant activity of lettuce performed better with a combination of blue light with a peak wavelength of 435 nm and red with a peak wavelength of 663 nm with a ratio of (1.25 ± 0.1), than with a combination of blue light with a peak wavelength of 450 nm and red with a peak wavelength of 663 nm with a ratio of (1.25 ± 0.1) at high intensity of (270 ± 20 µmol m^−2^ s^−1^). However, when a small amount of green light with a wavelength peak at 520 nm is added to the combination of B450/R663 nm and the ratio of B450 nm to Red663 nm is the same or R663/B450 nm = 1.2, all of the LEDs enhanced the assimilation rate by the same amount and produced the same amount of lettuce fresh weight. From these results, it can be concluded that B435/R at high intensity is the best LED for the production of economic yields of hydroponically grown lettuce in the plant factory for production of the highest yields. It was also found that B435/R at the low intensity of 60 µmol m^−2^ s^−1^ is the best LED for producing the highest level of antioxidant activity.

Grow LED light system is a growing area for both research and commercial applications. This will increase the capacity of testing more specific wavelengths in both red and blue regions of lights. This is currently is still one of the limitations for a deeper understanding of the plant response to light spectrum.

## Figures and Tables

**Table 1:** The effects of high light intensity of different LED treatments on A: Assimilation rate (for harvest stage (*p* ≤ 0.001), for light treatment (*p* = 0.003) for interaction between light treatments and harvest stage (*p* = 0.605)). B: Stomatal conductance (for harvest stage (*p* ≤ 0.001), for light treatments (*p* ≤ 0.001) and for interaction between light treatments and harvest stage (*p* ≤ 0.001)). C: transpiration rate LSD for harvest stage (*p* = 0.06) for treatments (*p* = 0.066), and for interaction light treatments and harvest stage (*p* = 0.001). D: Shoot fresh weight (g) (for harvest stages (*p* ≤ 0.001), for treatment (*p* = 0.003) and for interaction between harvest stage and light treatments (*p* = 0.08)). E: Root fresh weight (g) for harvest (*p* ≤ 0.001) for light treatments (*p* = 0.083) and interaction (*p* = 0.31). F: Leaves number (for harvest stage (*p* ≤ 0.001), for light treatment (*p* = 0.199) and for interaction between harvest stage and light treatments (*p* = 0.153)).

Light Treatment		Harvest Stage	Growth and Physiological Parameters					
			Assimilation Rate (μmol m^−2^ s^−1^)	Stomatal Conductance (mmol m^−2^ s^−1^)	Transpiration Rate (mmol m^−2^ s^−1^)	Shoot Fresh Weight (g)	Leaves Number	Root Fresh Weight (g)
Blue/Red (B435/R663)		Harvest stage 1	6.58 ± 0.31	0.5 ± 0.150	1.19 ± 0.021	84.9 ± 6.84	39.37 ± 4.30	8.6 ± 0.67
		Harvest stage 2	3.94 ± 0.33	0.38 ± 0.054	1.54 ± 0.106	165.30 ± 2.44	69.67 ± 6.09	11.62 ± 1
Blue/Red (B450/R663)		Harvest stage 1	5.03 ± 0.43	0.23 ± 0.024	1.20 ± 0.066	77.36 ± 8.83	25.25 ± 3.03	3.96 ± 0.87
		Harvest stage 2	1.95 ± 0.31	0.12 ± 0.012	1.23 ± 0.092	119.22 ± 2.45	67.83 ± 6.24	7.61 ± 0.64
Blue/Red treatment (B/R-rich)		Harvest stage 1	6.90 ± 0.47	1.09 ± 0.162	1.48 ± 0.074	60.31 ± 11.98	24.5 ± 4.52	4.22 ± 0.37
		Harvest stage 2	2.76 ± 0.36	0.20 ± 0.017	2.05 ± 0.126	137.01 ± 2.44	71.67 ± 8.86	8.83 ± 1.19
LSD	Harvest stage		2.41	0.40	Not significant	40.49	10.17	4.39
	Light treatment		1.96	0.33	1.25	33.06	Not significant	Not significant
	Interaction between light treatment and harvest stage		Not significant	0.23	0.70	Not significant	Not significant	Not significant

**Table 2:** The effects of low light intensity of different LEDs treatments on A: Assimilation rate (for harvest stage (*p* ≤ 0.001), for light treatments (*p* = 0.003), and for interaction between harvest stage and light treatments (*p* = 0.605)). B: Stomatal conductance (for harvest stage (*p* ≤ 0.001), for treatment (*p* = 0.072), and for interaction between harvest stage and light treatments (*p* ≤ 0.001)). C: transpiration rate (for harvest (*p* = 0.002) for light treatments (*p* = 0.003) and for interaction between harvest stage and light treatments (*p* = 0.31)). D: Shoot fresh weight (g) (for harvest stages (*p* = 0.001), for light treatments (*p* = 0.078), and interaction between harvest stage and light treatments (*p* = 0.069)). E: Root fresh weight (g) (for harvest (*p* ≤ 0.001) for light treatments (*p* = 0.013), and for interaction between harvest stage and light treatments (*p* = 0.210)), F: leaves number (for harvest stage (*p* ≤ 0.001), for light treatments (*p* = 0.07) for interaction between harvest stage and light treatments (*p* = 0.012)).

Light Treatment		Harvest Stage	Growth and Physiological Parameters					
			Assimilation Rate (μmol m^−2^ s^−1^)	Stomatal Conductance (mmol m^−2^ s^−1^)	Transpiration Rate (mmol m^−2^ s^−1^)	Shoot Fresh Weight (g)	Leaves Number	Root Fresh Weight (g)
Blue/Red (B435/R663)		Harvest stage 1	3.18 ± 0.64	0.16 ± 0.016	3.83 ± 0.81	29.6 ± 2.85	23.25 ± 2.37	7.57 ± 1.19
		Harvest stage 2	2.68 ± 0.62	0.09 ± 0.011	1.45 ± 0.1	75.87 ± 2.45	40.83 ± 3.81	10.18 ± 0.81
Blue/Red (B450/R663)		Harvest stage 1	2.44 ± 0.31	0.26 ± 0.019	2.38 ± 0.10	22.69 ± 3.27	21.5 ± 1.90	1.87 ± 0.22
		Harvest stage 2	1.04 ± 0.11	0.07 ± 0.007	0.93 ± 0.07	73.99 ± 2.45	46 ± 2.84	7.58 ± 0.61
Blue/Red treatment (B/R-rich)		Harvest stage 1	3.12 ± 0.37	0.16 ± 0.016	3.01 ± 0.69	26.62 ± 2.55	24.12 ± 2.41	3.79 ± 0.36
		Harvest stage 2	1.85 ± 0.30	0.11 ± 0.009	1.46 ± 0.10	44.98 ± 2.45	28.83 ± 3.92	9.05 ± 0.9
LSD	Harvest stage		2.41	0.073	0.54	30.19	5.06	2.55
	Light treatment		1.96	Not significant	0.44	Not significant	Not significant	2.08
	Interaction between light treatment and harvest stage		Not significant	0.04	0.31	Not significant	13.01	Not significant

**Table 3:** The effects of low light intensity of different LEDs treatments on A: Assimilation rate (LSD for harvest stages = 2.26 (*p* = 0.00), for treatments = no significance (*p* = 0.062), and for interaction = 1.31 (*p* = 0.00)). B: Stomatal conductance (LSD for harvest stages = no significance (*p* = 0.590), for treatments = 0.09 (*p* = 0.002), and for interaction = no significance (*p* = 0.118)). C: transpiration rate (LSD for harvest = 1.42 (*p* = 0.038), for treatments = 1.16 (*p* = 0.00), and for interaction = 0.82 (*p* = 0.010)). D: Shoot fresh weight (g) (LSD for harvest = 43.05 (*p* ≤ 0.001), for treatments = no significance (*p* = 0.598), and for interaction = no significance (*p* = 0.723)). E: Root fresh weight (g) (LSD for harvest = 3.35 (*p* = 00), for treatments = no significance (*p* = 0.86), and for interaction = no significance (*p* = 0.072)). F: Leaves number (for harvest stage (*p* ≤ 0.001), for light treatment (*p* = 0.277) for the interaction between harvest stage and light treatments (*p* = 0.044)).

Light Treatment		Harvest Stage	Growth and Physiological Parameters					
			Assimilation Rate (μmol m^−2^ s^−1^)	Stomatal Conductance (mmol m^−2^ s^−1^)	Transpiration Rate (mmol m^−2^ s^−1^)	Shoot Fresh Weight (g)	Leaves Number	Root Fresh Weight (g)
Blue/Red (B435/R663)		Harvest stage 1	3.30 ± 0.74	0.15 ± 0.01	1.58 ± 0.16	37.72 ± 2.72	21.62 ± 1.13	5.24 ± 1.04
		Harvest stage 2	2.76 ± 0.36	0.21 ± 0.017	2.05 ± 0.126	100.5 ± 2.45	46.5 ± 5.46	10.67 ± 0.57
Blue/Red (B450/R663)		Harvest stage 1	4.04 ± 0.37	0.19 ± 0.027	1.20 ± 0.066	33.81 ± 3.36	23 ± 2.84	1.89 ± 0.14
		Harvest stage 2	2.27 ± 0.27	0.15 ± 0.016	1.41 ± 0.085	95.33 ± 2.45	50.33 ± 4.66	6.04 ± 0.43
Blue/Red treatment (B/R-rich)		Harvest stage 1	6.39 ± 0.45	0.25 ± 0.010	3.84 ± 0.729	24.87 ± 4.79	24.75 ± 1.77	2.26 ± 0.51
		Harvest stage 2	2.05 ± 0.20	0.22 ± 0.020	1.89 ± 0.091	82.21 ± 2.45	36 ± 2.73	10.23 ± 1.04
LSD	Harvest stage		2.26	Not significant	1.42	43.05	5.30	3.35
	Light treatment		Not significant	0.09	1.16	Not significant	Not significant	Not significant
	Interaction between light treatment and harvest stage		1.31	Not significant	0.82	Not significant	14.71	Not significant
